# Public Health Discussions on Social Media: Evaluating Automated Sentiment Analysis Methods

**DOI:** 10.2196/57395

**Published:** 2025-01-08

**Authors:** Lisa M Gandy, Lana V Ivanitskaya, Leeza L Bacon, Rodina Bizri-Baryak

**Affiliations:** 1 Department of Computer Science College of Sciences and Liberal Arts Kettering University Flint, MI United States; 2 Department of Health Administration The College of Health Professions Central Michigan University Mt Pleasant, MI United States; 3 Department of Healthcare Management Northwood University Midland, MI United States

**Keywords:** ChatGPT, VADER, valence aware dictionary for sentiment reasoning, LIWC-22, machine learning, social media, sentiment analysis, public health, population health, opioids, drugs, pharmacotherapy, pharmaceuticals, medications, YouTube

## Abstract

**Background:**

Sentiment analysis is one of the most widely used methods for mining and examining text. Social media researchers need guidance on choosing between manual and automated sentiment analysis methods.

**Objective:**

Popular sentiment analysis tools based on natural language processing (NLP; VADER [Valence Aware Dictionary for Sentiment Reasoning], TEXT2DATA [T2D], and Linguistic Inquiry and Word Count [LIWC-22]), and a large language model (ChatGPT 4.0) were compared with manually coded sentiment scores, as applied to the analysis of YouTube comments on videos discussing the opioid epidemic. Sentiment analysis methods were also examined regarding ease of programming, monetary cost, and other practical considerations.

**Methods:**

Evaluation methods included descriptive statistics, receiver operating characteristic (ROC) curve analysis, confusion matrices, Cohen κ, accuracy, specificity, precision, sensitivity (recall), *F*_1_-score harmonic mean, and the Matthews correlation coefficient. An inductive, iterative approach to content analysis of the data was used to obtain manual sentiment codes.

**Results:**

A subset of comments were analyzed by a second coder, producing good agreement between the 2 coders’ judgments (κ=0.734). YouTube social media about the opioid crisis had many more negative comments (4286/4871, 88%) than positive comments (79/662, 12%), making it possible to evaluate the performance of sentiment analysis models in an unbalanced dataset. The tone summary measure from LIWC-22 performed better than other tools for estimating the prevalence of negative versus positive sentiment. According to the ROC curve analysis, VADER was best at classifying manually coded negative comments. A comparison of Cohen κ values indicated that NLP tools (VADER, followed by LIWC’s tone and T2D) showed only fair agreement with manual coding. In contrast, ChatGPT 4.0 had poor agreement and failed to generate binary sentiment scores in 2 out of 3 attempts. Variations in accuracy, specificity, precision, sensitivity, *F*_1_-score, and MCC did not reveal a single superior model. *F*_1_-score harmonic means were 0.34-0.38 (SD 0.02) for NLP tools and very low (0.13) for ChatGPT 4.0. None of the MCCs reached a strong correlation level.

**Conclusions:**

Researchers studying negative emotions, public worries, or dissatisfaction with social media face unique challenges in selecting models suitable for unbalanced datasets. We recommend VADER, the only cost-free tool we evaluated, due to its excellent discrimination, which can be further improved when the comments are at least 100 characters long. If estimating the prevalence of negative comments in an unbalanced dataset is important, we recommend the tone summary measure from LIWC-22. Researchers using T2D must know that it may only score some data and, compared with other methods, be more time-consuming and cost-prohibitive. A general-purpose large language model, ChatGPT 4.0, has yet to surpass the performance of NLP models, at least for unbalanced datasets with highly prevalent (7:1) negative comments.

## Introduction

The Pew Research Center [1] reports that as of 2021, 72% of Americans used social media. On a global scale, as of 2019, social media platforms were used by 1 in 3 people worldwide and by more than two-thirds of all internet users [2]. With social media users rapidly increasing, user-generated content has grown exponentially. Generated by a broad swath of global citizens, the data provides insights into a wide array of human experiences, for example, the effects of the opioid crisis on physical, mental, and social well-being.

Analysis of the opioid epidemic revealed the struggles of opioid victims, their families, and communities. This information adds value to health policy analysis.

This study examines the use of YouTube, a unique social media platform. It is a diverse medium because its primary purpose is content sharing and educating its users about relevant topics [3]. Users seek out content of interest and, comment and react. When examining health care crises and the relay of medical information, sentiment analysis may be used to gauge the response to the stimulus. By analyzing the sentiments expressed in comments, researchers can perform qualitative content analyses to explore how these reactions influence reputation, potentially affect individuals, and impact the communities involved. Furthermore, understanding the sentiment toward health-related content or issues like human suffering and societal concerns can aid policy makers in developing strategies to enhance public health.

Sentiment analysis is the most popular artificial intelligence used to mine and examine all text types in various fields of study. Sentiment analysis is a computational method that extracts sentiment from a text. Some sentiment analysis methods use rule-based lexicons such as Linguistic Inquiry and Word Count (LIWC)-22 [4]. Other sentiment analysis methods use traditional machine learning approaches such as Support Vector Machines [5] and Naive Bayes classification [6], while others use deep learning [7]. Sometimes, a hybrid approach is used between 2 or more sentiment analysis methods [8].

This study compares 3 popular sentiment analysis methods on social media data: Valence Aware Dictionary for Sentiment Reasoning (VADER), TEXT2DATA (T2D), and LIWC, and also uses the ChatGPT large language model (LLM) for sentiment analysis. A total of 2 methods (VADER and LIWC) were picked due to their previous validation and use in many published studies by scholars from different disciplines; other methods were chosen because of their user-friendly interface and no requirement of prerequisite programming skills (LIWC-22 and T2D). ChatGPT was chosen as LLM represent a game-changing technological leap in natural language processing (NLP), including sentiment analysis.

VADER [9] is a rule and lexicon-based sentiment analysis tool that, when analyzing text, returns a numeric valence (polarity) score between –1 (extremely negative) and +1 (extremely positive). The VADER lexicon is primarily built using pre-existing lexicons, but these lexicons were extended to include emoticons, acronyms, and slang commonly used in social media. Each lexical feature is assigned a score; then, the score is shifted based on the presence of punctuation marks, capital letters, and negations. The lexical scores are then averaged. VADER has been validated on multiple data types, including product and movie reviews, and has been used as a quality benchmark in numerous studies [9-11]. It has been used for sentiment analysis in a wide variety of areas, such as customer reviews and opinion mining [12,13], political discourse analysis [14,15], and mental health studies [16,17]. Although VADER has been used extensively, concerns about VADER’s sensitivity to text length have been expressed [18,19]. For example, as each word in a text is assigned a polarity score, words with strong sentiment can unduly influence the overall sentiment of a short text. There are proposed methods to mitigate this phenomenon [9], such as considering the text of an entire document about a sentence. Another possible issue is that VADER is primarily used as a Python-based program, and users must be moderately proficient in Python programming to use its full capabilities.

T2D [20] is a Microsoft Excel or Google Sheets add-in with a sentiment analysis application programming interface (API). It classifies text into 5 categories: very negative, negative, neutral, positive, and very positive. The T2D website contains scant information about the corpus or the methodology used for sentiment classification. The website states that the API is “based on an NLP engine” and that the “system also contains specially prepared classification models for Twitter (rebranded as X) and other social media content, trained on billions of manually verified entries” [20]. It is a paid subscription model, but users can make 1000 API calls a month for free. The next tier is 10,000 API calls for US $27 a month, then a variety of tiers exist, with the highest tier being Enterprise at US $351 a month and Unlimited API calls. As a Microsoft Excel add-in and a user-friendly tool for those familiar with Microsoft Excel, T2D has been applied to studies that examined tourist experiences [21], compared Twitter comments with polling results [22], and analyzed digital mental health interventions [23].

LIWC-22 is a dictionary-based sentiment analysis tool with a representative score in over 100 categories. The score in each category indicates the percentage of words in the text corresponding to the particular category and, therefore, can range from 0% to 100%. Many LIWC-22 categories are organized in a hierarchical structure. The same word may be categorized into multiple categories. For instance, “celebrate” is in both the positive emotion and achievement categories. Each category is represented internally in LIWC-22 by a dictionary with words and emoticons related to that category. A complete list of LIWC-22 categories can be found in [24]. The creators of LIWC-22 selected a vast pool of words that represented a wide range of linguistic categories and psychological dimensions, such as emotions, cognitive processes, social terms, pronouns, prepositions, and other linguistic constructs. LIWC-22 is the fifth iteration of LIWC-22 (LIWC 2001, 2007, 2015, and 2019). To create LIWC, experts in linguistics and psychology first gathered a large corpus of texts and tagged each word into a linguistic category. The resulting dictionaries underwent validation studies and then passed through a refinement phase when researchers added new vocabulary and, as meanings shifted, modified existing word-category pairings. LIWC-22 is a subscription model. Currently, users pay US $129.95 for a 3-year subscription, and shorter-term subscriptions are offered at lower prices. LIWC-22 has both a web-based app and an app for download. The users upload their file (JSON, CSV, and EXCEL), choose the LIWC-22 dictionary they would like to use, and then select the label in their data that denotes the text to be classified. Users also have the option to choose all or specific LIWC-22 categories to be evaluated.

Similar to VADER and T2D, LIWC-22 has been used as a sentiment analysis tool in many studies, such as education [25], public discourse analysis [26], and brand perception [27] among others. Many categories in LIWC-22 could represent sentiment, including positive emotion, negative emotion, anger, sadness, and anxiety. LIWC-22 includes positive tone and negative tone dimensions but also includes another Tone variable that merges the 2 dimensions into a single summary variable. The higher the number, the more positive the tone; numbers below 50 are counted as negative tone. This paper uses the LIWC-22 tone composite (summary) measure as its dictionary was more extensive than other sentiment-based LIWC-22 categories. We will refer to it as LIWC tone.

Previous efforts to evaluate sentiment analysis tools did not compare LIWC tone, VADER, and T2D. For example, Boukes et al [28] compared LIWC, SentiStrength, Pattern, Polyglot, and DANEW on economic news in Danish. These sentiment analysis tools were chosen as they were off the shelf and supported the Danish language. Hartmann et al [29] compared LIWC-22 with a host of machine learning-based sentiment analysis methods (Support Vector Machines, Neural Networks, K Nearest Neighbors, and Random Forests) but did not include VADER or T2D and focus on the marking aspect of social media data.

Recent advances in NLP have led to the development of powerful language models, such as the GPT series, including ChatGPT (GPT–3.5 and GPT–4) [30]. These models, pretrained on vast amounts of text data, have demonstrated strong performance across tasks like language translation, text summarization, and question-answering [31]. A LLM ChatGPT, in particular, has shown promise in education, health care, reasoning, text generation, human-machine interaction, and scientific research [30,32]. Despite these opportunities, challenges and ethical concerns remain, particularly regarding accuracy. The accuracy of these models depends heavily on the quality, diversity, and complexity of the training data, as well as the quality of user input [30,31]. Previous research has highlighted the importance of developing higher-order thinking skills in education, but NLP systems may struggle with the nuances of human language, leading to potential errors [30-32].

We compare the sentiment scores produced by VADER, T2D, LIWC_tone, and ChatGPT 4.0 to manually created codes. The data source is YouTube comments on videos that discussed the opioid epidemic. This data source was chosen as it was easily accessible to the authors, but also because each of the sentiment analysis classifiers mentioned has been heavily used to classify social media data in the past. As social media (and the data it creates) grows, researchers will likely analyze it using the sentiment tools discussed in this paper.

In the remainder of the paper, we discuss the methods by which data was collected and coded and will then compare the results given by VADER, T2D, LIWC_tone, and ChatGPT 4.0 to human coding. This paper reports which methods give accurate sentiment scores and discusses other practical considerations when using these tools. We will end with a discussion of the results and considerations for future research.

## Methods

### Data Sources

**Figure 1:**
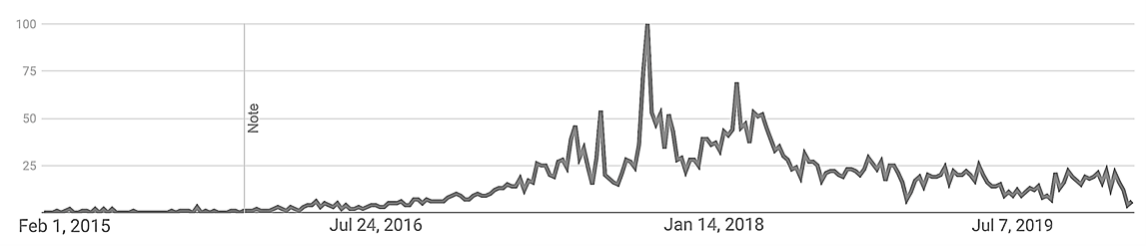
Google Trends results for the search term “opioid epidemic.” The search was constrained to dates between February 2015 and December 2019. The y-axis indicates percent popularity.

To evaluate sentiment analysis methods, we used secondary data from a qualitative study about the opioid crisis in the United States [33], which included manually coded sentiment from YouTube comments. To collect YouTube comments (N=8761), the term “opioid” epidemic was searched on YouTube with a date range between January 1, 2017, and December 31, 2018. The majority of the videos collected were located on CNN’s (Cable News Network) YouTube channel and the Fox News YouTube Channel. Subsequently, videos were ranked by the number of views, and the 20 most watched videos by CNN (10 newscasts) and Fox News (10 newscasts) were kept for further analysis. As Google Trends indicates (Figure 1), the chosen dates coincide with a particularly high interest in the opioid epidemic. The comments for each video were downloaded using the Netlytic website. Comments were deidentified by deleting email addresses and assigning codes such as a1, a2, and so on, to track comments on comments. All other comment information was left unaltered.

### Sentiment Measures

An inductive, iterative approach to content analysis of the data was used to obtain manual sentiment codes. Bacon [33] created a codebook to determine the sentiment of comments as positive, neutral, or negative based on the commenter’s attitude toward the video or another comment. The comments were sorted by time of post and video, then coded in the context of the broader discussion. Video transcripts also informed the coding. A subset of comments was analyzed by a second coder, producing good agreement between the 2 coders’ judgments (κ=0.734; 95% CI 0.57-0.89; *P*<.001).

To begin testing automated sentiment tools, comments with manual codes indicating neutral or unclear sentiment were excluded from the data. The remaining comments were scored using VADER, T2D’s Google Sheets add-in, LIWC, and ChatGPT 4.0. Specifically, the VADER compound score, T2D sentiment score, and LIWC-22 tone score (LIWC tone), a composite measure for positive and negative tone dimensions, were used. ChatGPT 4.0, VADER, and T2D were centered around 0 and ranged from –1 to 1. Negative values indicated a negative sentiment and positive values showed a positive sentiment. LIWC tone scores ranged from 1 to 100, representing percentiles based on standardized scores from large comparison corpora [34]. They are calculated using a dictionary with words, word stems, phrases, and select emoticons built for text analysis. LIWC tone overall mean for 15 diverse corpora was 47.81 (SD 26.39). Bacon’s codebook was adapted to prompt the general-purpose LLM to generate positive and negative sentiment classification (Multimedia Appendix 1 for the ChatGPT 4.0 prompt). The classification was generated on the third try after two failed attempts.

### Analyses

We performed the receiver operating characteristic (ROC) curve analysis and computed descriptive statistics, confusion matrices, Cohen , accuracy, specificity, precision, sensitivity (recall), *F*_1_-score harmonic mean, and the Matthews correlation coefficient (MCC). Relying solely on the ROC curve without considering precision and negative predictive value can lead to a misleading assessment of a model’s success [35]. Although widely used in machine learning, the *F*_1_-score also has limitations; it can vary when positive and negative classes are exchanged, potentially distorting its interpretation [36]. In addition, the *F*_1_-score does not account for correctly classified negative and positive samples, drawing criticism for diverging from more intuitive metrics like accuracy and losing significance when class labels are reversed [36]. In contrast, MCC provides a more balanced evaluation, achieving its highest values of –1 or +1 only when the classifier performs well across all 4 key rates of the confusion matrix: sensitivity, specificity, precision, and negative predictive value [35,36].

Finally, to reveal misclassification patterns by model, we analyzed the content of the comments marked as false positives and false negatives from both NLP and LLM models and compared them with true positives and true negatives identified through manual coding. We summarized possible reasons behind misclassifications and provided representative comments as illustrations.

### Ethical Considerations

Data were collected from a social media platform (YouTube) where data are publicly available. However, all sentiment evaluation methods were performed at a macro scale and not at the user level. In addition, social media profile information is not shared in the data provided in this article.

## Results

Manual coding placed 63.2% of comments into either positive or negative categories; the remainder were neutral, or their sentiment could not be ascertained. Only positively or negatively classified comments were used for further analyses (N=5533). Positive comments were much less common (79/662, 12%) than negative (4286/4,871, 88%) comments.

**Figure 2:**
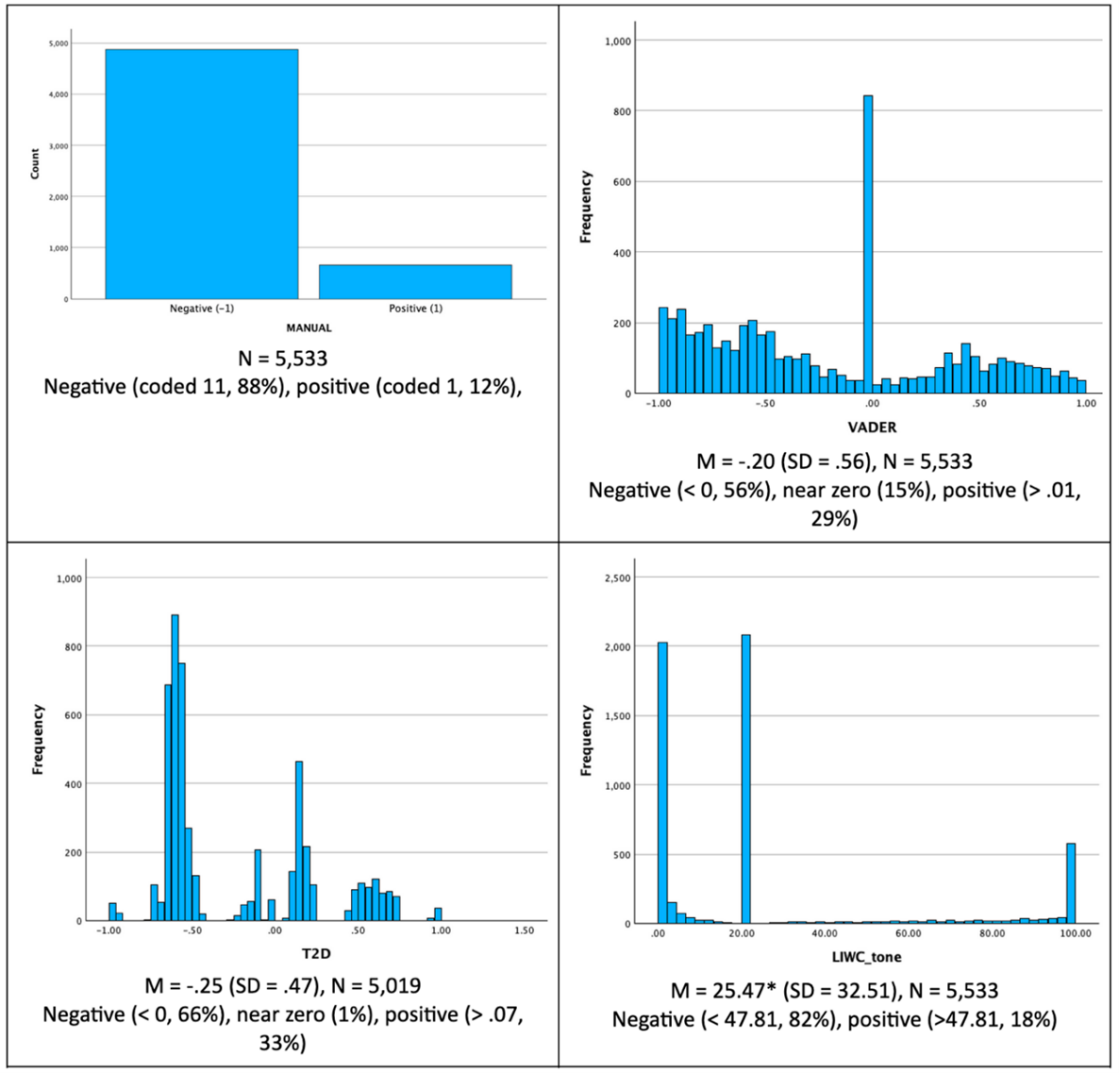
A comparison of Valence Aware Dictionary for Sentiment Reasoning, TEXT2DATA, and Linguistic Inquiry and Word Count tone score distributions for 5533 comments classified as either negative or positive using manual coding. *LIWC tone scores below 47.81 are considered negative. T2D: TEXT2DATA.

VADER, LIWC tone, and ChatGPT 4.0 were able to classify all comments. However, for unknown reasons, T2D sentiment scores could not be calculated for 514 comments, resulting in 9% of missing values. T2D is a “black box” system, and documentation has not been released. As shown in Figure 2, LIWC tone analysis most closely matched the high prevalence of negative comments (88%) in manually coded data: 82% of LIWC tone scores fell below the mean of 47.81. ChatGPT 4.0 overestimated negative sentiment, classifying 98% of the comments as negative. In comparison, only 56% of VADER and 66% of T2D scores were negatively scored. VADER score distribution had a mode around 2 and was the most continuous compared with T2D and LIWC. VADER assigned near-0 scores to 15% of comments, T2D had 1% of near-zero data but did not score 9% of comments, and LIWC tone scores were very unevenly distributed with 3 modes (at scale’s endpoints and 23.23).

As an overall measure of discrimination, we used the ROC curve [37]. Discrimination is the ability of a measure to discern between social media comments that are manually coded as negative or positive. We were interested in evaluating continuously measured VADER, T2D, and LIWC tone scores as predictors of positive and negative sentiment in YouTube comments; manual coding is an outcome against which the 3 models were assessed. ROC curve analysis was not performed for ChatGPT 4.0 because it was only generated as a binary measure. The area under the ROC curve is a measure of the overall discriminatory ability of the binomial logistic regression model, that is, the ability of a chosen sentiment scoring method to classify comments into the 2 groups of our dichotomous dependent variable, a manually coded sentiment where negative is assigned a value –1 and positive is given a value of 1. ROC curve analysis is most suitable for balanced analyses. While we are interested in correctly classifying positives and negatives, our dataset is unbalanced due to the high prevalence of negative comments.

**Figure 3:**
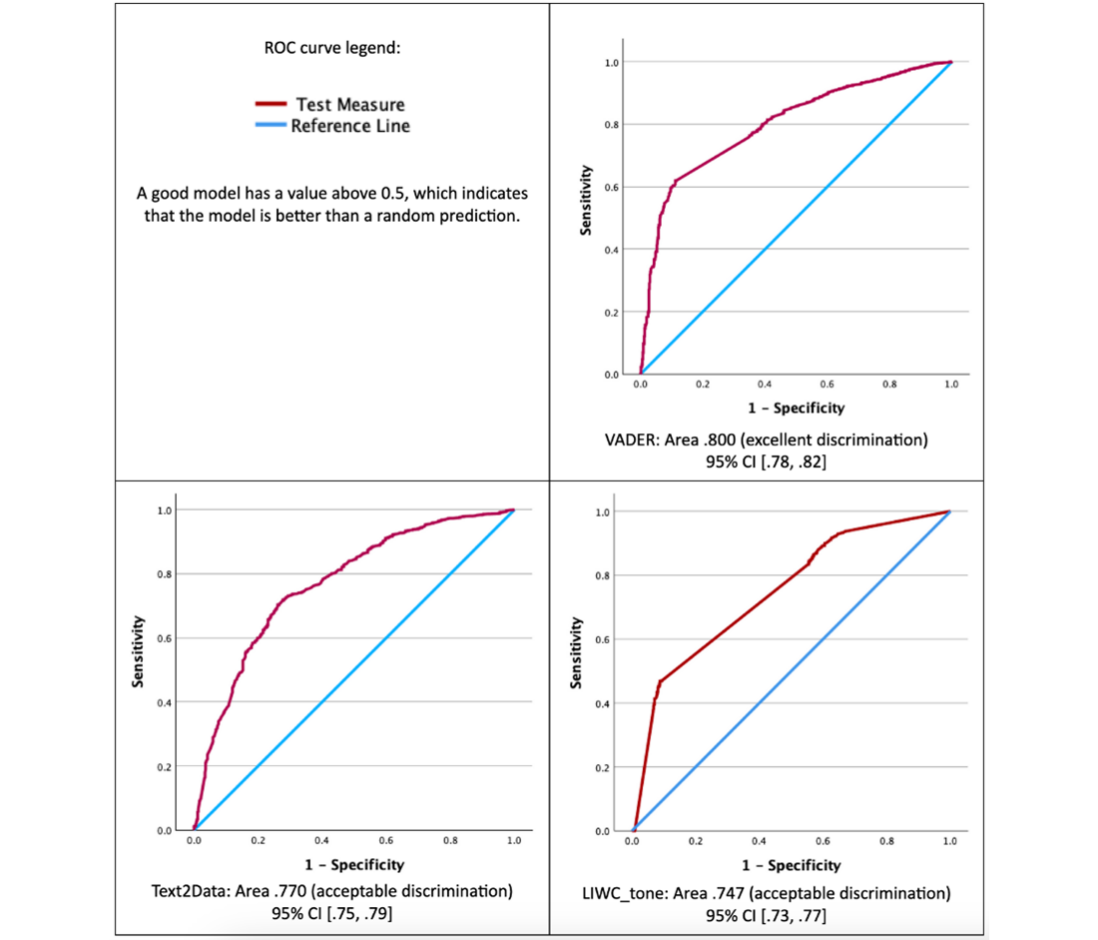
Receiver operating characteristic analyses: a comparison of Valence Aware Dictionary for Sentiment Reasoning, TEXT2DATA, and Linguistic Inquiry and Word Count tone scores’ ability to classify comments that were manually coded as either negative (–1) or positive (1).

As shown in Figure 3, ROC curves with red lines above the blue straight reference line indicate discrimination; the further above the reference line, the better. The area under the ROC curve is equivalent to the concordance probability [38]. Figure 2 shows an excellent level of discrimination, according to Hosmer et al [39], for VADER’s ability to classify manually coded negative comments (the area under the ROC curve was 0.800, 95% CI 0.78-0.82), followed by T2D and LIWC tone. T2D and LIWC tone demonstrated acceptable discrimination, with the areas under the curve of 0.770 and 0.747, respectively.

**Figure 4:**
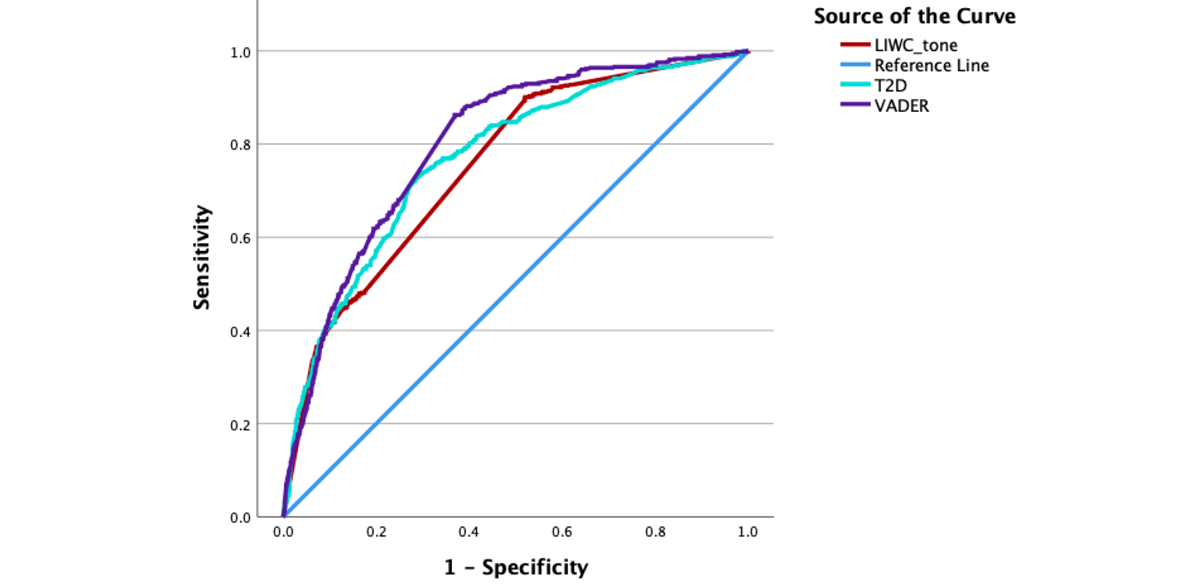
Receiver operating characteristic curve comparisons for Valence Aware Dictionary for Sentiment Reasoning, TEXT2DATA, and Linguistic Inquiry and Word Count Tone.

LIWC tone, according to Figure 4, performs the same or worse across the entire range of true positive rates (sensitivity), except at the short stretch of higher sensitivity rates where it is superior to T2D. Overall, VADER performs better than the 2 other sentiment analysis systems. Next, we tested if VADER performed better for longer strings of text. Figure 4 compares VADER results or all comments, regardless of length, and for longer comments (>100 characters and >200 characters long). VADER indeed performed better when short comments were excluded. Confusion matrices are given in Tables 1-4.

Table 5 shows 7 measures of model performance compared with manual coding that are Cohen , accuracy, specificity, precision, sensitivity (recall), *F*_1_-score, and MCC.

**Table 1:** A confusion matrix for binary coded Valence Aware Dictionary for Sentiment Reasoning, which are negative (–1) and positive (1) sentiments.

VADER^a^	Manual coding, n			Total, N
	–1	1		
–1	3693	230	3923	
1	1178	432	1610	
Total	3923	662	5533	

^a^VADER: Valence Aware Dictionary for Sentiment Reasoning.

**Table 2:** A confusion matrix for binary coded t2d: negative (–1) and positive (1) sentiment.

T2D^a^	Manual coding, n		Total, n
	–1	1	
–1.00	3218	145	3363
1.00	1277	379	1656
Total	4495	524	5019

^a^T2D: TEXT2DATA.

**Table 3:** A confusion matrix for binary coded LIWC_tone: negative (–1) and positive (1) sentiment.

LIWC_tone^a^	Manual coding, n			Total, n
	–1	1		
–1	4214	373	4515	
1	729	289	1018	
Total	4871	662	5533	

**^a^**LIWC: Linguistic Inquiry and Word Count.

**Table 4:** A confusion matrix for binary coded ChatGPT 4.0: negative (–1) and positive (1) sentiments.

ChatGPT 4.0	Manual coding, n, negative (–1); positive (1)			Total, n
	–1	1		
–1	4814	610	5424	
1	57	52	109	
Total	4871	662	5533	

**Table 5:** A comparison of Valence Aware Dictionary for Sentiment Reasoning, TEXT2DATA, Linguistic Inquiry and Word Count tone, and ChatGPT 4.0 against manual coding.

Model	Cohen κ (95% CI; *P* value)	Accuracy	Specificity	Precision	Sensitivity (recall)	*F*_1_-score	MCC^a^
VADER^b^	0.254 (95% CI 0.23- 0.28; <.001)	0.74	0.94	0.26	0.65	0.38	0.29
T2D^c^	0.225 (95% CI (0.2-.25; <.001)	0.72	0.96	0.23	0.72	0.35	0.3
LIWC^d^_tone	0.233 (95% CI 0.20-0.26; <.001)	0.80	0.92	0.28	0.47	0.34	0.24
ChatGPT 4.0	0.105 (95% CI 0.1-0.31; <.001)	0.88	0.88	0.50	0.08	0.13	0.16

^a^MCC: Matthews correlation coefficient.

^b^VADER: Valence Aware Dictionary for Sentiment Reasoning.

^c^T2D: TEXT2DATA.

^d^LIWC: Linguistic Inquiry and Word Count.

First, Cohen is used to examine the classification of YouTube comments into positive and negative sentiments. κ Values show fair agreement of manual coding with VADER (κ=0.254, 95% CI 0.23-0.28; *P*<.001), LIWC tone (κ=0.233, 95% CI 0.20-0.26; *P*<.001), and T2D (κ=0.26, 95% CI 0.20-0.25; *P*<.001). Overall, NLP demonstrated better agreement than LLM (κ=0.105, 95% CI 0.1-0.31; *P*<.001) for ChatGPT 4.0 indicating especially poor agreement of manual coding [40].

Second, ChatGPT 4.0 achieved the highest accuracy score of 88%, followed by LIWC tone (80%), VADER (74%), and T2D (72%). Third, the proportion of accurately detected negatives or specificity varied from 96% for T2D to 94% for VADER, 92% for LIWC tone, and 88% for ChatGPT 4.0. Fourth, precision reveals how well a model accurately makes positive predictions [41]. Precision values indicate that positive predictions were unlikely to be very accurate: ChatGPT 4.0 was at 50%, as compared with even lower values for LIWC tone (28%), VADER (26%), and T2D (23%).

Fifth, sensitivity, recognized as recall, detects true positives within a confusion matrix, with T2D achieving 72%, VADER achieving 65%, LIWC tone reaching 47%, and ChatGPT 4.0 achieving only 8% [41]. Sixth, the *F*_1_-score is the harmonic mean of precision and sensitivity (recall), offering an evaluation of true positives and positive predictive values was below 50% for all models: VADER (at 0.38) was closely followed byT2D (0.35), LIWC tone (0.34), whereas ChatGPT’s *F*_1_-score stood out as the lowest (0.13). Finally, MCC was calculated by measuring the sensitivity, specificity, precision, and negative predictive value to evaluate the model performance [36]. None of the MCCs reported in Table 5 reached a strong correlation level; they varied from 0.30 (T2D) to 0.29 (VADER), 0.24 (LIWC tone), and the lowest value of 0.16 for ChatGPT 4.0.

Large discrepancies observed between the automated classifications and human coding may be explained by specific linguistic features, contextual nuances, model limitations, and other factors. To uncover any underlying patterns contributing to misclassifications and determine whether specific errors are more prevalent in 1 model over another, we analyzed comments classified as false positives and false negatives by LLM and NLP models.

Table 6 shows how sentiment misclassification varied by model. None of the models stood out as being excellent at avoiding both false positives and false negatives; however, ChatGPT 4.0 performed especially poorly. It classified even the most obviously positive comments as negative sentiment, leading to a very high rate (92%) of false negatives.

An analysis of false positives did not produce model-specific patterns. Sarcastic comments expressing feigned empathy for drug users were frequently misidentified by all models as false positives, for example, when the commenters talked about people dying but also added phrases such as “*White addiction, i truly love it. Now lets see what happens. Lmbao!*” [comment ID: 756]. Other common false positives involved discussions about marijuana as a gateway drug, criticisms of both Democrats and Republicans, racist remarks, and the attribution of blame to drug users.

Across all 4 models, discussions about the legalization of marijuana were misclassified as false negatives, as illustrated by this comment,

The opioid crisis was huge here in Florida. Look at what happened to it when they legalized marijuana for medical purposes, opioid deaths dropped! Look at the states that have it as recreational, opioid use and crime drop 20% in just the first year!

comment ID: 3795

Our examination of false negatives also revealed model-specific, thematic patterns. VADER tended to misclassify political posts and comments on drug legalization as false negatives. T2D had difficulties with mentions of safe injection sites and support for Donald Trump. LIWC tone was prone to misclassifying short comments, those with emojis, as well as references to kratom, CBD (Cannabidiol) oil, and marijuana.

Finally, Table 7 summarizes the observed differences between sentiment analysis methods applied to our unbalanced social media dataset.

As shown in Table 7, T2D operates as a black box with minimal technical documentation, posing challenges for academic analysis. Unlike the other methods, VADER requires a moderate level of programming skill. All methods have low or no monetary cost (VADER is free) except for T2D, which charges per transaction, potentially making it cost-prohibitive for large datasets. Based on our findings, Table 7 provides other important considerations when selecting a model.

**Table 6:** Misclassified comments by model: false positives, false negatives, and representative comments.

Method, compared with manual coding	False positives, %	False negatives, %	Representative comments
VADER^a^	30.02	34.74	FP^b^: *Doctor’s have become legalized dope dealers, they tried to get my 17 nephew to take opioids. They gave him a 2 month supply. Luckily he didn’t take it because he’d seen what it can do. He’s still playing football no thanks to the doc and big pharma* [comment ID: 70]; FN^c^: *Yes! It [kratom] got me off pain meds... pain medication* [comment ID: 2470]
T2D^d^	28.40	27.67	FP: *Big Phama! Big Insurance! Doctors get a cut for each pill script filled! We’re worth more dead, than alive*! [comment ID 71]; FN: *Wish this war on opioids started earlier so many people gone. Very grateful for President Trump* [comment ID: 1483]
LIWC^e^_tone	14.97	56.34	FP: *Sue the opioid companies? What like the CIA^f^? LMFAO #CNNISFAKENEWS* [comment ID: 5872]; FN: K*ratom is the best maintenance program you could ever discover. Was on oxy for years, then methadone for years, then I found Kratom. It helps with pain, cravings, and has a bonus effect of reducing the craving for alcohol...* [comment ID: 2478]
ChatGPT 4.0	1.17	92.14	FP: *This isn’t helping anyone. The medical industry is already terrified to prescribe these drugs, you people who don’t take opioids have no idea of what damage you are doing to your medical system until you need to use it…* [comment ID: 909]; FN: *I have used MJ In the past to get off of Opioids and Xanax and Vicodin.. Yes, it does work and it also helps with Pain very well. It should be LEGAL even for NonMedical Use! It helps MANY problems and Various Ailments!* [comment ID: 3071]

^a^VADER: Valence Aware Dictionary for Sentiment Reasoning.

^b^FP: false positive.

^c^FN: false negative.

^d^T2D: TEXT2DATA.

^e^LIWC: Linguistic Inquiry and Word Count.

^f^CIA: Central Intelligence Agency.

**Table 7:** Considerations for selecting sentiment analysis methods when using social media datasets that are unbalanced toward negatives.

Method	Implementation type	Programming skill	Cost	Additional cons	Additional pros
Manual coding	Coding by a trained researcher	None	Time for creating codebook, manual coding	Retraining for intercoder reliability	Reaches good agreement when 2 humans code comments, accurate coding of ambiguous comments (sarcasm, etc)
VADER^a^	Rule-based dictionary	Moderate	Free	Not user-friendly for beginners, may code sarcastic comments as false positives.	Low runtime, fair agreement with manual codes, excellent discrimination, and performance can be improved by excluding short comments (<100 characters)
T2D^b^	Black box	None	Paid API^c^: Free up to 1000 transactions, US $27/ month for 10,000 transactions.	Might not code all data, implementation is not defined (black box), may code sarcastic comments as false positives.	Low learning curve, self-contained within the same spreadsheet, high runtime, fair agreement with manual codes, acceptable discrimination
LIWC^d^_tone	Rule-based dictionary across multiple LIWC dimensions	None	Academic license: US $55 (1-year license) US $129 (3-year license)	May misclassify sarcastic comments as false positives and short comments as false negatives.	Includes a contextualizer that highlights words in reported dimensions, low learning curve, low runtime, accurate estimation of prevalence of negative versus positive sentiment, fair agreement with manual codes, acceptable discrimination
ChatGPT 4.0	Large language model	None	US $20/month	Requires prompt design, might not be consistent between iterations, may not be responsive due to high API usage, poor agreement with manual codes, inaccurate estimation of prevalence of negative vs positive sentiment, low MCC^e^ may code sarcastic comments as false positives, hallucination possible.	Low runtime through the OpenAI API can provide reasoning for classification

^a^VADER: Valence Aware Dictionary for Sentiment Reasoning.

^b^T2D: TEXT2DATA.

^c^API: application programming interface.

^d^LIWC: Linguistic Inquiry and Word Count.

^e^MCC: Matthews correlation coefficient.

## Discussion

### Principal Findings

This study involved a comparison of manual sentiment coding to 4 automated sentiment analysis methods, namely VADER, T2D, LIWC_tone, and ChatGPT 4.0. We aimed to assess the efficacy of these sentiment analysis techniques in categorizing comments as either positive or negative sentiment in YouTube comments.

YouTube and other social media platforms are valuable repositories of comments and reviews on topics relevant to various organizations and stakeholders, such as businesses, public policy analysis, and politicians. Our corpus, comments on the US opioid crisis, was manually analyzed to reveal the struggles of opioid epidemic victims, their families, and communities, the issues of value to health policy analysis [33]. Like many other datasets of interest to social media researchers, it was skewed toward negative sentiment (7:1) and contained relatively few positive comments. Social media discussions often lean in the negative direction, such as Facebook posts on vaccine hesitancy [42] or long Covid discussions on Twitter (rebranded as X) [43]. Moreover, identifying social media content with negative effects is particularly valuable because it is more likely to be shared than positive content [44,45].

Our analysis showed that accurate positive sentiment classification can be important and policy-relevant when applied to negatively skewed data. The discussion of the opioid epidemic was primarily about human suffering but it was not all negative. Some YouTube users, for example, praised kratom as a means of overcoming opioid addiction. A subset of comments highlighted positive experiences of overcoming addiction and mixed reactions to health policies, such as safe injection sites and marijuana legalization. Marijuana, for example, can be discussed as a gateway drug (negative sentiment) and also from a harm reduction perspective as a substitute for opioids and other harmful street drugs (positive sentiment). Classifying the data by sentiment enables researchers to explore diverse perspectives of the digital public, potentially leading to health policy insights. Given the importance of sentiment analysis in unbalanced datasets, this study offers valuable guidance for social media researchers on the pros and cons of several available methods.

Overall, VADER performed best on the ROC curve analysis, demonstrating excellent discriminatory capabilities compared with LIWC tone (acceptable) and T2D (acceptable). VADER’s performance improved when short comments were excluded, a finding that verified VADER’s sensitivity to text length suggested by Nair et al [18] and Tymann et al [19]. However, the ROC analysis has limitations when applied to data with an unbalanced count of negative and positive comments. Also, it could not be computed for binary ChatGPT 4.0 data. LIWC tone performed better than other automated models when estimating the prevalence of negative and positive sentiment in our data.

A comparison of Cohen κ values indicated that the NLP models (VADER, followed by LIWC and T2D) showed only fair agreement of manual coding, whereas ChatGPT 4.0 had poor agreement. While all models performed better than chance when predicting the dominant class, leading to higher precision, their level of agreement with manual coding is not exceptionally high. Moreover, variations in accuracy, specificity, precision, and sensitivity (recall), *F*_1_-score, and MCC did not suggest a single superior model. All models evaluated had relatively low *F*_1_–scores, which serve as overall prediction performance measures that combine precision and recall. *F*_1_-score was below 50% for all automated models and especially low for ChatGPT 4.0. The same pattern was observed for MCC: none of the models resulted in a significant correlation (T2D’s value was the highest at 0.30), but ChatGPT 4.0 performed the worst. While we cannot endorse a particular best model, our analysis suggests caution when using ChatGPT 4.0 to classify sentiment in an unbalanced dataset.

A total of 5 sets of instructions were uploaded to train ChatGPT 4.0 to conduct sentiment analysis, incorporating the codebook used by manual coders. The file upload process could have been more convenient, and 2 out of 3 attempts resulted in errors during file reading. LLM sentiment analysis was only accessible through a paid subscription, as the free version could not handle uploading and coding thousands of comments or formatting the results for further statistical analysis and comparison. Despite this, ChatGPT 4.0, like NLP models, seemed to rely on identifying words labeled as positive or negative in the codebook to classify comments, which might have led to misclassifications of sarcastic comments.

ChatGPT 4.0 outperformed the NLP models as an LLM in only 2 measures: accuracy and precision. It required specific coding instructions expressed as prompts, which we derived from the manually created codebook. Even though the same codebook was used for manual coding and designing ChatGPT 4.0 prompts, their level of agreement could have been better. Considering generation failures, poor agreement with manual coding, and the need for a paid subscription, ChatGPT 4.0 may be different from the model of choice for social media researchers looking to perform sentiment analysis on unbalanced datasets. Our comparison of ChatGPT 4.0 to 3 NLP models indicated that the general-purpose LLM has yet to surpass the performance of traditional NLP models, at least for unbalanced datasets with highly prevalent (7:1) negative comments.

Across all models, false negatives were associated with discussions on the legalization of marijuana and the observed reduction in mortality in states with more permissive drug policies. The primary issue with NLP models may stem from their reliance on pre-existing dictionaries to classify sentiment in a way that is not target specific. They cannot interpret the nuances of certain words within specific contexts the way a human can. To mitigate this, LIWC-22 and similar NLP models may require the creation of tailored dictionaries to better grasp the particular meanings of words in relevant contexts. Even then, NLP models may never be able to differentiate between negative statements of fact and negative sentiment with a specific target.

Not only NLP models but perhaps also LLM, tended to link any drug-related vocabulary with negative sentiment, failing to consider the context or nuance, particularly in discussions about overcoming the opioid crisis. This highlights a significant limitation of dictionary-based NLP models: their inability to accurately classify positive comments or recognize positive aspects of complex, contentious discussions compared with the accuracy of manual coding. Misclassification was observed for comments with sarcasm, leading models to mistake feigned empathy for genuine concern. Sentiment classification is complex and requires a deep understanding of the issues to interpret social media discourse accurately.

Manual coding remains the most reliable method for detecting sentiments when analyzing complex topics on social media, especially for unbalanced datasets. In addition to being time-intensive, it has other limitations. According to Krippendorff [46], texts often have multiple meanings. Manual coding is an interpretive process that may only sometimes match the commenter’s intent, even when there is a good interrater agreement. On the other hand, automated sentiment classification may not consistently align with human judgments due to sentiment’s inherently subjective and context-dependent nature, lowering reliability [47]. Fair reliability can be considered appropriate in cases involving complex or subjective tasks, exploratory research, resource constraints, qualitative analysis, or research contexts where a high level of agreement is not a primary objective [47]. Researchers must carefully weigh the trade-offs between achieving higher reliability and the practical constraints specific to their research.

Future research should explore whether general-purpose and fine-tuned LLMs and NLP models demonstrate comparable discriminatory performance in social media samples where positive comments are more prevalent than negative ones. Researchers should also test different methods for prompting general-purpose LLMs to follow complex manual codebook instructions.

### Conclusions and Recommendations

We offer suggestions for VADER, T2D, LIWC tone, and ChatGPT 4.0 applications in the semantic classification of social media, specific to an unbalanced dataset with a high prevalence of negative comments. None of the automated models emerged as a clear leader. With caution, we recommend a no-cost tool, VADER, due to its excellent discrimination, according to our ROC curve analysis, which improves when the comments are at least 100 characters long. VADER requires some programming skills and may underestimate the prevalence of negative comments in unbalanced datasets. LIWC tone may be useful for social media researchers studying negative emotions, public worries, or dissatisfaction when they need to accurately estimate the prevalence of positive versus negative comments in their datasets. Researchers using T2D must know that it may only score some data and, compared with other NLP methods, can be time-consuming and cost prohibitive. ChatGPT 4.0 did not demonstrate superior performance. While the use of general-purpose LLMs is promising, it remains to be determined how to translate manual codebook instructions into prompts best to achieve superior classification results.

## Data Availability

A codebook with representative comments has been added through Multimedia Appendix 1, as well as a copy of the ChatGPT 4.0 prompts used in sentiment analysis, and a list of videos that evoked YouTube comments.

## Appendix

Multimedia Appendix 1 Representative code book and the prompt used for ChatGPT sentiment analysis. A list of videos from which YouTube comments were extracted are also included.

## Abbreviations

API: application programming interface
LIWC: Linguistic Inquiry and Word Count
LLM: large language model
MCC: Matthews correlation coefficient
NLP: natural language processing
ROC: receiver operating characteristic
T2D: TEXT2DATA
VADER: Valence Aware Dictionary and Sentiment Reasoning
