# Supplementary material for: Public Health Discussions on Social Media: Evaluating Automated Sentiment Analysis Methods
Source: JMIR Form Res. 2025 Jan 8;9:e57395. doi: 10.2196/57395 (PMC11784633; doi:10.2196/57395)
Supplement: Multimedia Appendix 1 [file formative_v9i1e57395_app1.pdf]

Multimedia Appendix

1. Sentiment Classification Guide with Representative Negative Comments

Sentiment Classification	Comment
Negative	Death penalty for drug dealers will equal death penalty for cops. Enforcement will become very dangerous because if they are going to die then they will take cops with them.
Negative	How about the fact that he’s busy cutting down his own base by hitting healthcare in this country, eliminating subsidies for things like airports in rural communities, doing nothing about the opioid crisis OR proper hurricane relief for Puerto Rico OR the ongoing mental health crisis?
Negative	I don't think he knows amthing about opioids. Maybe he thinks Mexicans are growing them and walking them over. So dumb
Negative	The word drug dealer is a dog whistle term that his stupid idiot base of bigots associate with people of color.
Negative	They're too stupid to know that opioids are the creation of pharmaceutical companies, so the drug dealers would be doctors writing these prescriptions for profit. I have COPD fibromyalgia and a very bad heart condition.
Negative	I believe it is my choice if I want to take opioids klonopin Xanax anything that's going to bring me relieve and if I die in the process of that that too as in my Rite! Do not regulate people or the doctors they are our only chance to lead a halfway normal life
Negative	FDA-AMA etc. are Glorified Drug Dealers for Big-Pharma---FDA legally prescribed opioids are pharmaceutical grade HEROIN produced in a laboratory -- -!!!
Negative	you must be an opioid crack head user..
Negative	IT IS ONLY AN OPIOID EPIDEMIC WHEN WHITE PEOPLE GET ADDICTED, BUT WHEN BLACKS AND LATINOS IS USING DRUGS THERE IS NOT EPIDEMIC. OPIOID BEEN AROUND BEFORE MOST OF US WAS BORN. IT DID NOT LEAVE AND THEN CAME BACK. SINCE THE MIDDLE CLASS IS AFFECTED BY DRUGS IT IS AM EPIDEMIC.

2. Sentiment Classification Guide with Representative Positive Comments

Sentiment Classification	Comment
Positive	Thank you President Trump for knowing they are real people with pain.
Positive	I'm sorry to hear this! I know the president is only trying to do the right thing. Hopefully there will be a good resolution to your needs, my prayers are with all who deal with chronic pain.
Positive	Great news!!! Way to go!! Never go back, never give up!! ❤
Positive	good for you...we do recover....November 7years for me.I also quit smoking cigarettes in December I feel so good and I'm in my early 50s...
Positive	Whatever you're doing is working so keep on keeping on 😎🎃🕷🙏🏼🙏🏼
Positive	THANK YOU MR. PRESIDENT!
Positive	I believe we can fix this problem thank you for addressing this, helping our country to be a better place and helping to create a better future!
Positive	What President Trump is doing against Opioids is so much appreciated. This is a WONDERFUL thing.
Positive	My family I went through hell because of addiction. Altered our lives forever. Thank you so much President Trump. It’s about time!
Positive	I have some beautiful ideas that really help with sustainable recovery with addiction. I just need help with funds to accomplish it. Please respond for more information. Facebook.com/searchedandrescued

Multimedia Appendix

1. Instructions to ChatGPT 4.0 for Classifying Positive and Negative Sentiment

- Code each comment separately
- Code for positive and negative sentiment
- Code positive sentiment as 1.00
- Code negative sentiment as -1.00
- Provide the coding in a spreadsheet format
- Provide the comment ID number and the positive or negative sentiment spreadsheet
- Use positive and negative sentiment examples to guide the classification

2. Examples of comments classified by manual coding as negative sentiment for ChatGPT 4.0

- Death penalty for drug dealers will equal death penalty for cops. Enforcement will become very dangerous because if they are going to die then they will take cops with them.
- 736k How about the fact that he's busy cutting down his own base by hitting healthcare in this country, eliminating subsidies for things like airports in rural communities, doing nothing about the opioid crisis OR proper hurricane relief for Puerto Rico OR the ongoing mental health crisis?
- 1071k I don't think he knows amthing about opioids. Maybe he thinks Mexicans are growing them and walking them over. So dumb
- 1136k - The word drug dealer is a dog whistle term that his stupid idiot base of bigots associate with people of color.
- They're too stupid to know that opioids are the creation of pharmaceutical companies, so the drug dealers would be doctors writing these prescriptions for profit. I have COPD fibromyalgia and a very bad heart condition.
- I believe it is my choice if I want to take opioids klonopin Xanax anything that's going to bring me relieve and if I die in the process of that that too as in my Rite! Do not regulate people or the doctors they are our only chance to lead a halfway normal life.
- FDA-AMA etc. are Glorified Drug Dealers for Big-Pharma---FDA legally prescribed opioids are pharmaceutical grade HEROIN produced in a laboratory -- -!!!
- 802 you must be an opioid crack head user..
- IT IS ONLY AN OPIOID EPIDEMIC WHEN WHITE PEOPLE GET ADDICTED, BUT WHEN BLACKS AND LATINOS IS USING DRUGS THERE IS NOT EPIDEMIC.

OPIOID BEEN AROUND BEFORE MOST OF US WAS BORN. IT DID NOT LEAVE AND THEN CAME BACK. SINCE THE MIDDLE CLASS IS AFFECTED BY DRUGS IT IS AM EPIDEMIC.

3. Examples of comments classified by manual coding as positive sentiment for ChatGPT 4.0

- Thank you President Trump for knowing they are real people with pain.
- I'm sorry to hear this! I know the president is only trying to do the right thing. Hopefully there will be a good resolution to your needs, my prayers are with all who deal with chronic pain.
- Great news!!! Way to go!! Never go back, never give up!! 🖤
- 527 good for you...we do recover....November 7years for me.I also quit smoking cigarettes in December I feel so good and I'm in my early 50s...
- Whatever you're doing is working so keep on keeping on 😎🎃🕷🙏🏼🙏🏼
- THANK YOU MR. PRESIDENT!
- I believe we can fix this problem thank you for addressing this, helping our country to be a better place and helping to create a better future!
- What President Trump is doing against Opioids is so much appreciated. This is a WONDERFUL thing.
- My family I went through hell because of addiction. Altered our lives forever. Thank you so much President Trump. It’s about time!
- I have some beautiful ideas that really help with sustainable recovery with addiction. I just need help with funds to accomplish it. Please respond for more information. Facebook.com/searchedandrescued

4. Response from ChatGPT 4.0 containing python code for classifying sentiment in a spreadsheet format

```
# Re-importing necessary library

import pandas as pd

# Load the newly uploaded CSV file

file_path_latest = '/mnt/data/Comments-VADER scores for validation.csv'

data_latest = pd.read_csv(file_path_latest)

# Display the first few rows of the dataset to understand its structure

data_latest.head()

❑IL
```

Video Titles and YouTube Upload Dates for 10 Most Viewed CNN Newscasts, Ranked by the Number of Views

Rank	CNN Video Title	YouTube Upload Date
1	Van Jones rips Trump’s death penalty for drug dealers proposal	3/19/18
2	Trump tells Sessions to sue certain opioid companies	8/18/18
3	Trump declares opioids a national emergency	10/26/17
4	Could marijuana be the solution to the opioid crisis?	4/27/18
5	Philadephia’s controversial plan to fight opioid epidemic	10/20/18
6	Can this plant fix the opioid crisis?	10/26/17
7	White House: Trump donated salary check to opioid crisis	11/30/17
8	Price: Trump treating opioid crisis as emergency	8/8/17
9	Conway defends WH opioid epidemic plan	6/23/17
10	Gov. Christie compares opioid deaths to 9/11	7/31/17

Video Titles and YouTube Upload Dates for 10 Most Viewed Fox News Videos, Ranked by the Number of Views

Rank	Fox News Video Title	Date
1	‘Drugged’: Big Pharma, the FDA, and the opioid crisis	4/11/17
2	Trump delivers remarks, signs opioid bill at the White House	10/24/18
3	‘Drugged’: Inside the opioid crisis	4/10/17
4	Inside the worst drug-induced epidemic in US history	4/10/17
5	The dark web’s connection to the opioid epidemic	4/13/17
6	Will carfentanil cause the next opioid epidemic in the US?	4/19/18
7	Can marijuana help fight the opioid epidemic?	4/4/18
8	Medical industry an accomplice in opioid epidemic	3/2/18
9	How serious is the opioid epidemic?	10/17/17
10	Opioid crisis: What will Trump’s ‘public health emergency’ declaration actually do?	10/26/17
